# Antiangiogenic agents in advanced gastrointestinal malignancies: past, present and a novel future

**DOI:** 10.18632/oncotarget.187

**Published:** 2010-10-15

**Authors:** Karen Mulder, Sheryl Koski, Andrew Scarfe, Quincy Chu, Karen King, Jennifer Spratlin

**Affiliations:** Medical Oncology, Cross Cancer Institute, University of Alberta, Alberta, Canada

**Keywords:** angiogenesis, anti-angiogenic agents, Bevacizumab, sorafenib, gastrointestinal cancers, biomarkers

## Abstract

Advanced gastrointestinal (GI) malignancies are varied in presentation, prognosis, and treatment options. With the exception of resectable recurrent colorectal cancer, metastatic GI malignancies are incurable. Cytotoxic chemotherapies have been the mainstay of therapy for decades but limited extension of survival or clinical benefit has been achieved in non-colorectal GI cancers. There has been great interest in the incorporation of antiangiogenic strategies to improve outcomes for these patients. Clear benefits have been identified with bevacizumab and sorafenib in colorectal cancer and hepatocellular cancer, respectively; other GI tumor sites have lacked impressive results with antiangiogenic agents. In this review, we will present the benefits, or lack thereof, of clinically tested antiangiogenic compounds in GI malignancies and explore some potential new therapeutic anti-angiogenesis options for these diseases.

## INTRODUCTION

**Figure 1:**
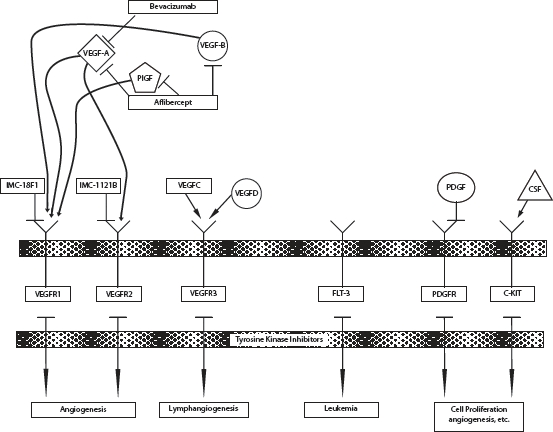
Vascular endothelial growth factor pathway adapted from QC previous paper Abbreviations: VEGF, vascular endothelial growth factor; VEGFR, vascular endothelial growth factor; PDGF, platelet derived growth factor ; PDGFR, platelet derived growth factor receptor; PlGF, platelet derived growth factor

Up-regulation of angiogenesis is required for development of malignancy, tumor growth and progression [1,2]. The vascular endothelial growth factor family of ligands (VEGF-A (VEGF), VEGF-B, VEGF-C, VEGF-D, VEGF-E, and placental growth factor(PlGF)) and receptors (VEGFR-1, VEGFR-2, VEGFR-3, FLT-3, platelet derived growth factor receptor (PDGFR), and C-KIT) are the most studied angiogenic pathways [3,4]. (FIGURE 1) Extracellular interaction of the ligands with VEGFRs encourages receptor dimerization, leads to receptor autophosphorylation, and subsequently activates downstream angiogenic and growth pathways. VEGF binds primarily to VEGFR-1 and VEGFR-2 whose expression is more pronounced in tumor vasculature endothelial cells [5]. VEGF/VEGFR binding results in cellular proliferation, vascular differentiation, altered vascular permeability, and migration [6-13].

Inhibiting cellular angiogenic machinery is a well researched area of cancer care. Over the last decade, several antiangiogenic compounds have been developed, investigated, and approved for cancer treatment. Inhibiting angiogenesis can occur via various mechanisms. To date, drug development has focused on blocking this pathway via inhibition of the ligand (VEGF), the receptors (VEGFRs), and the effector intracellular tyrosine kinase pathways. (TABLE 1) When the addition of bevacizumab, a monoclonal antibody (mAb) inhibiting VEGF, to chemotherapy for advanced colorectal cancer (CRC) offered an almost 5 month survival benefit, considerable effort was directed to develop other angiogenesis blockers[14]. Subsequent studies have attempted to match this benefit in other tumor types.

**Table 1:** Comparison of anti-angiogenic agents Abbreviations: VEGF, vascular endothelial growth factor; VEGFR, vascular endothelial growth factor; PDGF, platelet derived growth factor ; PDGFR, platelet derived growth factor receptor; PlGF, platelet derived growth factor; CRC, colorectal cancer; HBC, hepatobiliary cancers; HTN, hypertension; DVT, deep venous thrombosis; CHF, congestive heart failure; PE, pulmonary embolism; ALT, alanine transferase; HFS, hand-foot syndrome; LVEF, left ventricular ejection fraction.

Anti-angiogenic agent	Mechanism of Action	Tumour Sites of Interest	Toxicities from dose finding studies
Bevacizumab	IV recombinant humanized monoclonal antibody against VEGF	CRC	Anemia, dyspnea, intracranial bleed, tumor hemorrhage
Sunitinib	Oral multitargeted TKI inhibiting VEGFR-1, VEGFR-2, PDGFR-β, c-KIT, FLT3, and RET	HBC	Fatigue, HTN, bullous skin toxicity, elevated lipase/amylase, decrease LVEF, edema, thrombocytopenia, tumor necrosis, asthenia, nausea, vomiting, HTN, PE, phlebitis, neutropenia, thrombocytopenia, skin toxicity, tumor related fistulas, anemia
Sorafenib	Oral multitargeted TKI inhibiting VEGFR-1, VEGFR-2, PDGFR-β, Raf-1, B-Raf, and intracellular serine-threonine kinases	HBC	Rash, HTN, dyspnea, fatigue, HFS, abdominal cramping, diarrhea, retrosternal pain, edema of uvula, anorexia, fatigue, anorexia, diarrhea, rash/desquamation, HFS, nausea, alopecia
Cediranib	Oral TKI to VEGFR-1, VEGFR-2, VEGFR-3	Gastric CRC	Hypertension, hypertensive crisis, hypoglycemia, elevated bilirubin, fatigue, diarrhea, nausea, dysphonia, hypertension, vomiting, anorexia
Valatinib	Oral TKI to VEGFR-1, VEGFR-2, VEGFR-3, PDGFR, c-kit	CRC	Lightheadedness, fatigue, vomiting, nausea, diarrhea, HTN
Brivanib	Oral TKI to VEGFR-2 and FGFR-1	CRC	HTN, fatigue, AST/ALT elevation, thyroid dysfunction, hyponatremia
ZD6474	Oral TKI to VEGFR-2, RET, Flt-4, VEGFR-3, EGFR	pancreas	Diarrhea, HTN, rash, folliculitis, hypophosphatemia, increased ALT, bowel obstruction, colitis, fatigue, thrombocytopenia, rash, nausea, HTN, fatigue, anorexia, CHF, PE, DVT, bowel ischemia
ABT-869	Oral TKI to VEGFR-1, VEGFR-2, VEGFR-3, PDGFRß, and Flt3	CRC HCC	Fatigue, proteinuria, HTN, asthenia, HFS, myalgia
Ramucirumab	IV anti-VEGFR2 mAB	CRC	HTN, DVT, headache, proteinuria, vomiting, amylasemia
IMC-18F1	IV anti-VEGFR1 mAB	CRC	Fatigue, nausea, anemia
Aflibercept	VEGF-A, VEGF-B, PIGF fully human recombinant decoy fusion protein	gastric pancreas CRC	Rectal ulceration, proteinuria, HTN

Advanced gastrointestinal (GI) malignancies are a wide spectrum of diseases with variable prognoses depending on the stage at diagnosis. On one end of this spectrum is advanced gastric and pancreatic cancers which have uniformly poor overall survival from diagnosis regardless of treatment; on the other end of this spectrum is metastatic CRC which, when treated with a sequence of well established therapies, affords a median survival in excess of two years. The use of biologic agents, particularly antiangiogenics, has been at the forefront of clinical investigations in GI malignancies for most of the last decade. Mechanistically, the theories behind inhibiting new blood vessel formation, including improved delivery of concurrently administered cytotoxic drugs and promotion of effective delivery of blood and nutrients intra-tumorally, are valid. Unfortunately, for uncertain reasons, many antiangiogenic agents have not been effective in GI cancers. Herein, we review the use of antiangiogenic compounds which have proven clinical benefit in GI malignancies, discuss some novel agents currently under investigation, and make critical conclusions as to the effectiveness of this avenue as anti-cancer treatment in GI tumors

## ESOPHAGOGASTRIC CANCERS (EGC)

Gastric and esophageal cancers are the second and sixth leading causes of cancer related death worldwide, respectively [15]. Esophageal adenocarcinoma is now more prevalent than squamous cell carcinoma in North America and Western Europe and, as it is often difficult to determine if the cancer originates in the gastroesophageal junction or distal esophagus, patients with advanced diseases are often treated akin to advanced gastric adenocarcinoma. There is little data on the role of angiogenesis inhibitors in patients with esophageal squamous cell carcinoma. As such, this discussion will be limited to EGC adenocarcinoma.

Patients with metastatic EGC have 5 year survival rates of 10-15%. When compared to best supportive care, palliative cytotoxic chemotherapy improves survival [16-18]. Although there is no single internationally accepted standard of care regimen, the best survival rates are achieved with three drug combinations compared to doublet therapy [19].

VEGF and angiogenesis appear to have an important role in pathogenesis and prognosis of EGC. VEGF expression increases with increasing stage and disease burden in EGC [20,21]. Furthermore, VEGF expression is a negative prognostic factor for survival in this patient group [22,23]. In a gastric xenograft model, inhibition of VEGF activity by an immuno-neutralizing antibody was effective suggesting that VEGF inhibition may have therapeutic value [24]. Phase II studies of bevacizumab combined with chemotherapy (irinotecan and cisplatin; oxaliplatin and docetaxel or 5-fluoruracil (FU); docetaxel, cisplatin, and FU) showed promising results in previously treated and untreated patients (RR 63-71%) [25-28]. The phase III study, AVAGAST, which combined bevacizumab or placebo with capecitabine and cisplatin showed a significant improvement in overall response rate (ORR 38% vs 29.5%) and progression free survival (PFS 6.7 vs 5.3 months) [29]. However, the study failed to improve overall survival (OS), its primary endpoint.

Ramucirumab, a monoclonal antibody directed at VEGFR-2, is currently being tested in a randomized phase III study as a second-line agent in metastatic EGC (NCT009117384).

Several small molecule TKIs to VEGFRs have also undergone early phase II testing in EGC. Sorafenib in combination with docetaxel and cisplatin in treatment-naive patients with metastatic EGC demonstrated partial responses (PR) of 41%, median PFS of 5.8 months, and median OS of 13.6 months [30]. Sunitinib as a second-line single agent treatment for advanced gastric cancer demonstrated a disease control rate of 35% [31].

## CANCERS OF THE HEPATOBILIARY TRACT

### Hepatocellular Carcinoma (HCC)

HCC is the third leading cause of death worldwide after lung and gastric cancer [32]. Less than 30% of patients are eligible for surgery due to advanced stage of disease at presentation and treatment with cytotoxic chemotherapy has been disappointing with multiple studies failing to show an improvement in OS [33]. HCC's are highly vascular tumors. High microvessel density and levels of circulating VEGF are associated with poorer outcomes, thus making the angiogenesis pathway an attractive therapeutic target [34-39].

Sorafenib is the first systemic agent demonstrating an improvement in OS in patients with advanced HCC. The initial phase II study of 137 patients showed promising activity with a median OS of 9.2 months and a median time to progression (TTP) of 5.5 months [40]. Patients with Child-Pugh Class B liver function had similar incidence of drug-related adverse events but had more frequent worsening of liver disease (encephalopathy 11% vs. 2%; worsening ascites 18% vs. 11%) than patients with Child-Pugh A liver function as well as significantly worse OS (14 weeks vs. 41 weeks) [41]. Two phase III, multicenter, randomized, placebo-controlled studies confirmed the activity of this agent. Both studies limited enrollment to patients with Childs-Pugh A liver function. The SHARP study enrolled patients from Europe, North and South America and Australasia and had hepatitis C and alcohol as the predominant risk factors for HCC. The Asia-Pacific trial enrolled patients from China, South Korea and Taiwan and had hepatitis B as the predominant HCC risk factor. Both studies demonstrated a significant OS improvement (SHARP: 10.7 vs. 7.9 months, HR 0.69, p<0.001; Asia-Pacific: 6.5 vs. 4.2 months, HR 0.68, p=0.014) and disease control rate (SHARP: 43% vs. 32%, p=0.0002; Asia-Pacific: 35.5% vs. 15.8%, p=0.0019) with sorafenib as compared to best supportive care. Response rates (RR) were low (2% and 3.3% respectively) and there was no difference between the arms in time to symptomatic progression [42,43].

In early phase II studies, sunitinib also demonstrated activity in the treatment of advanced HCC [44,45]. However, a phase III study comparing sunitinib to sorafenib was terminated in April 2010 due to increased toxicity in the sunitinib arm; it did not meet the pre-defined criteria for superiority or non-inferiority (NCT00699374).

Two phase II studies examining the activity of single-agent bevacizumab in advanced HCC both demonstrate promising antitumour activity (RR 12.5-13%; PFS 6.9 months) but toxicity, in particular gastrointestinal bleeding, is concerning, [46,47]. There have been three single arm phase II studies of bevacizumab in combination with a variety of chemotherapy regimens which show evidence of clinical activity but randomized comparisons are required [48-50].

### Biliary tract cancers (BTC)

BTC, which include intra- and extra-hepatic cholangiocarcinoma and gallbladder malignancies, are rare tumours accounting for only 3-4% of GI cancers. Surgery is the only curative option but most patients present with unresectable disease [51]. In patients with advanced BTC, only recently has treatment with gemcitabine and cisplatin demonstrated an improvement in OS [52].

In contrast to HCC, BTC metastases tend to be hypovascular. However, VEGF expression in these tumors does correlate with advanced disease stage and poor prognosis [53,54]. A phase II clinical trial using gemcitabine and oxaliplatin (GEMOX) combined with bevacizumab demonstrated modest activity with an ORR of 40%, median PFS 7.0 months and median OS of 12.7 months [55]. However, the 6-month PFS did not meet the pre-specified endpoint of an improvement from 50% to 70% as compare to GEMOX alone. Randomized comparisons are needed to evaluate the added benefit of bevacizumab. To date, attempts at TKI inhibition have not been beneficial in BTC. Two phase II clinical trials of sorafenib failed to show significant clinical activity [56,57].

## PANCREATIC ADENOCARCINOMA

Pancreas cancer is the fourth commonest cause of cancer-related mortality across the world, with incidence equaling mortality [58]. Only 15-20% of patients present with surgically resectable disease and, of these, only 20% will survive 5 years. The OS for patients with metastatic or locally advanced disease is 4-9 months. Gemcitabine remains the standard chemotherapy for this disease with a modest benefit in OS [59]. Multiple studies combining gemcitabine with other cytotoxic agents have not demonstrated improvement in survival [60-62]. The phase III randomized trial of gemcitabine vs gemcitabine + erlotinib, an epidermal growth factor receptor (EGFR) inhibitor did improve median OS from 5.91 months to 6.24 months but the clinical relevance of this benefit is questioned by the medical oncology community [63].

VEGF and its receptors (VEGFR-1 and VEGFR-2) are co-expressed in pancreatic cancer suggesting that VEGF could have autocrine effects on pancreatic cancer cells that express VEGF receptors and paracrine effects on microvascular endothelial cells [64-66]. In animal models, VEGF TKIs and anti-VEGF and anti-VEGFR-2 antibodies inhibit growth and angiogenesis associated with pancreatic tumors and potentiated the tumoricidal effect of gemcitabine [67-71].

Activity in the phase II trials combining gemcitabine and bevacizumab looked promising and, based upon these results, bevacizumab was tested in two randomized controlled phase III studies [72,73]. In these trials, gemcitabine with or without bevacizumab and gemcitabine plus erlotinib with or without bevacizumab both failed to show a survival benefit with the addition of bevacizumab [74,75]. Other VEGF targeted therapies tested in the setting of pancreatic cancer include sorafenib, axitinib, and sunitinib but limited activity in phase II trials ended further investigations into these agents [76-78].

## COLORECTAL ADENOCARCINOMAS

Colorectal cancer is the fourth most common cancer in men and women [79]. Patients with untreated metastatic CRC (mCRC) have a median survival of 5 – 6 months [80]. Prior to the emergence of the topoisomerase I inhibitor irinotecan in 1996, treatment for metastatic colorectal cancer was limited to FU and leucovorin (LV). This was followed in 2004, by the introduction of oxaliplatin. An analysis of several large, phase III studies demonstrates that exposure at some time during treatment to these three agents (FU plus LV, irinotecan, and oxaliplatin) significantly improves overall survival OS of mCRC to an average of 20 months [81].

In early pre-clinical work on colon cancer specimens, a correlation was noted between increased VEGF expression and proliferative activity in tumors [82]. Furthermore, *in vivo* murine antihuman monoclonal antibodies targeted against VEGF inhibited growth of human tumor xenografts [83]. Based on these findings, two randomized phase II studies combining FU +LV with bevacizumab demonstrated improved clinical efficacy over FU + LV alone [84,85]. (Table 2) The landmark phase III trial by Hurwitz *et al* which led to the clinical approval of bevacizumab for the treatment of mCRC compared either FU + LV + irinotecan (IFL) + bevacizumab to IFL + placebo which reported a median OS of 20.3 months versus 15.6 months [14]. (Table 2) However, the IFL regimen has fallen out of favor due to the improved tolerance and efficacy of infusional FU + irinotecan (FOLFIRI) as compared to modified IFL (mIFL) [86]. This trial was then modified in April 2004 to assess the addition of bevacizumab to both of these arms of the trial with both the PFS and median OS favoring the patients receiving FOLFIRI + bevacizumab as compared to mIFL + bevacizumab. Although this trial did not directly compare FOLFIRI administered with or without bevacizumab, it does suggest FOLFIRI + bevacizumab is more efficacious than mIFL + bevacizumab.

**Table 2:** Seminal publications supporting the use of bevacizumab in advanced colorectal cancer Abbreviations: HR, hazards ratio; OR, odds ratio.

First-Line Phase II Trials	Relevant Treatment Arms	*n*	Response Rate		Progression-Free Survival		Overall Survival	
AVF0780g	Roswell Park	36	17%	Not applicable	5.2 months	Not applicable	13.8 months	Not applicable
	Roswell Park + Bevacizumab 5 mg/kg	35	40%		9.0 months		21.5 months	
	Roswell Park + Bevacizumab 10 mg/kg	33	24%		7.2 months		16.1 months	
AVF2192g	Roswell Park + Placebo	105	15.2%	Not applicable	5.5 months	Not applicable	12.9 months	Not applicable
	Roswell Park + Bevacizumab	104	26.0%		9.2 months		16.6 months	
**First-Line Phase III Trials**	**Relevant Treatment Arms**	***n***	**Response Rate**		**Progression-Free Survival**		**Overall Survival**	
AVF2107g	IFL	411	34.8%	*p* = 0.004	6.2 months	HR 0.54 *p* < 0.001	15.6 months	HR 0.66 *p* < 0.001
	IFL + B	402	44.8%		10.6 months		20.3 months	
BICC-C	FOLFIRI	144	47.2%		7.6 months		23.1 months	
	mIFL	141	43.3%		5.9 months		17.6 months	
	CapeIRI	145	38.6%		5.8 months		18.9 months	
	FOLFIRI + Bevacizumab	57	57.9%		11.2 months		Not yet reached	
	mIFL + Bevacizumab	60	53.3%		8.3 months		19.2 months	
NO16966	FOLFOX/XELOX + Placebo	699	38%	OR 1.00 *p* = 0.99	9.4 months	HR 0.83 *p* = 0.0023	21.3 months	HR 0.89 *p* = 0.0769
	FOLFOX/XELOX + Bevacizumab	701	38%		8.0 months		19.9 months	
**Second-Line Phase III Trials**	**Relevant Treatment Arms**	***n***	**Response Rate**		**Progression-Free Survival**		**Overall Survival**	
E3200	FOLFOX4	285	8.6%	*p* < 0.0001	4.7 months	HR 0.61 *P* < 0.0001	10.8 months	HR 0.75 *p* = 0.001
	FOLFOX4 + Bevacizumab	287	22.7%		7.3 months		12.9 months	

The combination of bevacizumab with oxaliplatin-based chemotherapy as first line therapy has also been investigated in a randomized, double-blind study designated N016966 [87]. In this study, 1,400 patients received FU + oxaliplatin (FOLFOX) or capecitabine + oxaliplatin (XELOX), with either bevacizumab or placebo. Although an improvement in median PFS was seen, neither an improvement in RR or median OS was achieved. (Table 2) It is hypothesized that the failure to improve median OS was due to early discontinuation of the capecitabine or FU and bevacizumab when oxaliplatin peripheral neurotoxicity occurred hence diminishing the impact of bevacizumab [88]. In the second-line setting, the addition of bevacizumab to FOLFOX improved RR, median PFS, and median OS [89]. (Table 2)

The clinical efficacy of bevacizumab in the metastatic setting led to the development of two pivotal phase III clinical trials in patients with resected stage II or III colon cancer. The NSABP C-08 study failed to show an improvement in disease-free or overall survival with the addition of bevacizumab to FOLFOX [90]. The results of the second trial, AVANT, are anticipated later this year (NCT00112918).

Several oral angiogenesis inhibitors are under investigation for the treatment of mCRC. (Table 1) Agents which have completed phase III clinical trials include valatinib and cediranib. Valatinib inhibits all known VEGF tyrosine kinase receptors. There have been two phase III studies testing this agent in mCRC. In CONFIRM-1, patients were assigned to receive first-line FOLFOX + valatinib or placebo but this study failed to meet its primary endpoint of PFS [91]. Similarly, CONFIRM-2, which was the second-line study of FOLFOX + valatinib or placebo, also did not meet its primary endpoint for OS [92]. HORIZON III is a randomized comparison of FOLFOX + cediranib (AZ2171), a highly potent and selective inhibitor of the three VEGF receptors, and FOLFOX + bevacizumab as first-line chemotherapy in mCRC. Although not yet published, a media release in March 2010 has confirmed that the study failed to meet its primary endpoint of non-inferiority for PFS [93].

## PROMISING NOVEL ANTIANGIOGENIC AGENTS FOR GI TUMORS

Despite many clinical trials, the only approved antiangiogenic therapies in GI tumours are bevacizumab and sorafenib, in advanced CRC and HCC, respectively. These will be the gold standard that all other agents in their class must compete against and no others have yet been successful. Additionally, there are no approved indications in gastroesophageal and pancreatic cancers for antiangiogenic compounds. Nonetheless, multiple other novel agents are currently under investigation. Below is a summary of the most promising agents that may prove beneficial in patients with GI malignancies.

## SMALL MOLECULE TKIS

### Brivanib alaninate

Brivanib alaninate is an oral small molecule TKI active against VEGFR-1, VEGFR-2, VEGFR-3, and fibroblast growth factor (FGF) receptor (FGFR). The FGF pathway has a demonstrated role in cancer progression and FGF level are elevated akin to VEGF levels in malignancy [94,95]. Dual blockade of VEGFRs and FGFR is attractive clinically as FGF signaling has been implicated in resistance to VEGFR inhibition [96]. There is particular interest in the development of this drug in some GI malignancies due to particularly high levels of FGF in HCC and the known FGF overexpression in gastric cancer [97,98]. Multiple phase I studies have demonstrated safety of brivanib both alone and in combination with cytotoxics. Ongoing trials in GI cancers are listed in table 3.

**Table 3:** Ongoing clinical trials with anti-VEGF TKIs Abbreviations: GI, gastrointestinal; HCC, hepatocellular; N/A, not applicable; Bev, bevacizumab; CRC, colorectal cancer; GEJ, gastroesophageal junction.

NCT Trial Number	Phase	Tumor Type	Line of therapy	Control arm	Investigational arm
NCT01046864	I	GI, not pancreas	N/A	N/A	5FU/LV+brivanib; FOLFIRI+brivanib FOLFIRI+brivanib in Japanese
NCT00825955	III	HCC	2^nd^	BSC+placebo	BSC+brivanib
NCT01108705	III	HCC (asian)	2^nd^	BSC+placebo	BSC+brivanib
NCT00640471	III	CRC	≥ 3^rd^	cetuximab+placebo	cetuximab+brivanib
NCT00858871	III	HCC	1^st^	sorafenib+placebo	brivanib+placebo
NCT00437424	I	HCC with liver dysfxn	N/A	N/A	brivanib
NCT00594984	I/II	CRC	N/A	N/A	Irinotecan+cetuximab+brivanib
NCT00355238	II	HCC	≥ 1^st^	N/A	brivanib
NCT00207051	I	Advanced GI	N/A	N/A	cetuximab+brivanib
NCT00707889	II	CRC	≥ 2^nd^	FOLFOX+Bev	FOLFOX+high dose ABT-869; FOLFOX+low dose ABT-869
NCT00517920	II	HCC	N/A	N/A	ABT-869
NCT01009593	III	HCC	1^st^	sorafenib	ABT-869
NCT00753675	II	biliary	1st	gemcitabine+placebo	AZD6474+placebo; AZD6474+gemcitabine
NCT00500292	II	CRC	2^nd^	FOLFOX+placebo	FOLFOX+low dose AZD6474; FOLFOX+high dose AZD6474
NCT00508001	II	HCC	1^st^	BCS+placebo	BSC+ low dose AZD6474; BSC+high dose AZD6474
NCT00454116	II	CRC	2^nd^	FOLFIRI+placebo	FOLFIRI+low dose AZD6474; FOLFIRI+high dose AZD6474
NCT00436072	I	CRC		N/A	cetuximab+AZD6474; irinotecan+cetuximab+AZD6474
NCT00681798	I	Pancreas	any	N/A	gemcitabine+capecitabine+AZD6474
NCT00732745	I/II	Esophagus GEJ	Any	docetaxel+oxaliplatin	docetaxel+oxaliplatin+AZD6474
NCT00499850	I	CRC	Any	N/A	FOLFOX+AZD6474
NCT00532909	I	CRC	any	N/A	capecitabine+oxaliplatin+cetuximab+AZD6474
NCT00683787	II	gastroesop	≤ 2^nd^	docetaxel	docetaxel+low dose AZD6474; docetaxel+high dose AZD6474

### ABT-869

ABT-869 is an oral potent TKI inhibitor of VEGFR-1, VEGFR-2, VEGFR-3, PDGFRß, and Flt3 [99,100]. Data suggests ABT-869 is more selective toward VEGFR and PDGFR than other similar TKIs while also having apoptotic effects [101]. Efficacy was seen with this drug in colorectal xenografts models [101]. A phase I dose escalation study recently published reported drug tolerability with toxicities including proteinuria, hypertension, fatigue, hand-foot blistering, and myalgias [99]. Three patients achieved partial response with 48% of patients recording stable disease. Effective antiangiogenesis was noted on dynamic contrast enhanced magnetic resonance imaging (DCE-MRI) which may act as a biomarker after further evaluation. The majority of clinical development of this agent is ongoing in CRC and HCC. Interim results from a phase II study in Child-Pugh A and B HCC reported tolerable toxicities and 42% of evaluable patients were progression free at 16 weeks [102]. Other ongoing GI trials using ABT-869 are listed in table 3.

### ZD6474

ZD6474 is a TKI potently inhibiting VEGFR-2, Rearranged during Transfection (RET), Flt-4, and EGFR [103,104]. Initial dose finding studies in patients with advanced solid malignancies who had failed standard of care treatments report good tolerability at doses <300mg [105,106]. As preclinical data supported use in a wide spectrum of human malignancies, clinical development of AZ6474 has been across multiple tumor types. In colon cancers, ZD6474 has been combined with FOLFIRI and FOLFOX with good tolerability [107,108]. A phase II study of FOLFIRI plus ZD6474 did not confirm benefit with the combination [109]. A combination of gemcitabine + capecitabine + ZD6474 in biliary and pancreatic cancer has shown promising early clinical results [110]. There is additional preclinical data supporting ZD6474 use in gastric cancers [111,112]. Ongoing clinical trials in GI cancers are listed in table 3.

## INHIBITING VEGFRS

### Ramucirumab

Ramucirumab is a fully human mAb with low pM affinity to the VEGF-binding domain of VEGFR-2 [113]. Dose-finding studies were undertaken with weekly and every 2 and 3 week single agent administration with very good drug tolerance [114,115]. Common toxicities were as expected based on the antibodies' mechanism of action and included thrombotic events, hypertension, proteinuria, and bleeding. Initial reports suggest a greater clinical benefit with the use of ramucirumab than other clinically tested antiangiogenic agents, though randomized clinical trials must be undertaken to prove superiority [114,116]. More advanced studies using a dose of 8 mg/kg every 2 weeks are ongoing and listed in table 4. Early data from the phase II study of ramucirumab used in the first-line setting in Childs-Pugh A and B HCC is promising in sorafenib-naïve patients with a 50% disease control rate [117].

**Table 4:** Ongoing clinical trials with anti-VEGFR mAbs Abbreviations: HCC, hepatocellular; CRC, colorectal; BSC, best supportive care; Bev, bevacizu.

NCT Trial Number	Phase	Tumor Type	Line of therapy	Control arm	Investigational arm
NCT01140347	III	HCC	2nd	BSC	ramucirumab
NCT01170663	III	gastric	2nd	paclitaxel+placebo	paclitaxel+ramucirumab
NCT01183780	III	CRC	2nd	FOLFIRI+placebo	FOLFIRI+ramucirumab
NCT01079780	II	CRC	≥ 2^nd^, post Bev	irinotecan+cetuximab	Irinotecan+cetuximab+ramucirumab
NCT01111604	II/III	CRC	2nd	FOLFOX	FOLFOX+ramucirumab; FOLFOX+IMC-18F1
NCT00917384	III	gastric	2ND	placebo+BSC	Ramucirumab+BSC

### IMC-18F1

IMC-18F1 has had a slower clinical development compared to ramucirumab. An intravenous recombinant human IgG_1_ anti-VEGFR-1 mAb, IMC-18F1 inhibits ligand-induced VEGFR-1 activation at low pM concentrations [118]. Final data from a multi-schedule phase I single agent dose finding study is pending but preliminary information revealed safely and biologic activity supporting further development of the drug [119]. Clinical development of IMC-18F1 is listed in table 4.

### Decoy fusion proteins

Aflibercept, a novel fully human recombinant decoy fusion protein, has recently completed phase I clinical testing [120,121]. Mimicking immunoglobin domains of VEGFR-1 and VEGFR-2, it has low pM affinity for VEGF, VEGF-B and PlGF [122,123]. When administered intravenously every 2 weeks, aflibercept was well tolerated with rectal ulceration and proteinuria being the dose limiting toxicities. Other toxicities were in keeping with inhibition of the VEGF/VEGFR pathway. Three patients experienced a partial response. A phase II study in metastatic CRC has also demonstrated benefit in a bevacizumab pre-treated cohort prompting further investigations in this area [124]. Ongoing studies with aflibercept in GI malignancies are listed in table 5.

**Table 5:** Ongoing clinical trials with aflibercept Abbreviations: CRC, colorectal cancer; 5FU, %-fluorouracil; N/A, not applicable.

NCT Trial Number	Phase	Tumor Type	Line of therapy	Control arm	Investigational arm
NCT00574275	III	pancreatic	2^nd^	placebo	aflibercept
NCT00561470	III	advanced CRC	2^nd^	irinotecan + 5FU	irinotecan+5FU+aflibercept
NCT00851084	II	advanced CRC	1^st^	FOLFOX	FOLFOX+aflibercept
NCT00407654	II	advanced CRC	± 2^nd^	N/A	aflibercept
NCT00921661	I	advanced CRC in Japanese	N/A	N/A	FOLFIRI+aflibercept

## SUMMARY

Antiangiogenic agents have clearly advanced the treatment of GI malignancies, most notably CRC and HCC. Unfortunately, there have been no advances in the treatment of incurable EGC and pancreas cancer using antiangiogenic agents and prognosis remains poor. Despite the promising results in CRC with bevacizumab and HCC with sorafenib, multiple clinical trials using other methods of blocking the VEGF pathway have been negative in these and the other GI malignancies. The mechanisms of resistance to VEGF inhibition is not known. Despite preclinical models suggesting that VEGF inhibition should be effective in all GI malignancies; this has not been confirmed in clinical practice.

Uncertainty exists in many of these negative trials as to why the study or drug failed. Likely, the answer is multifactorial being related to a combination of drug ineffectiveness once used in a randomized phase III setting, poor clinical trial design, suboptimal patient selection, and lack of a reliable biomarker to direct clinicians on which patients will or will not benefit. The importance of predictors for treatment response of these agents are exemplified well by the results of the AVAGAST study suggesting that there is a patient population which may benefit from the addition of an angiogenesis inhibitor, but when studied in a general patient population, a survival benefit could not be attained. Biomarkers are needed to delineate those patients who will benefit and those who will not.

In CRC, investigations are underway to determine genetic profiles or other patient characteristics to help identify those who will benefit from anti-angiogenic inhibition. Until trials can clarify the optimal use of these agents, data supports exposure, at some point in the treatment of mCRC, to bevacizumab in fit patients. In all GI tumors, the use of newer genetically engineered cancer models may help elucidate mechanisms of resistance or biomarkers to aid clinician treatment choices [125]. Additionally, though not necessarily practical, the use of newer imaging techniques, like DCE-MRI, may play a role in early determination of antiangiogenic efficacy.

Until the time when biomarkers are identified and validated to predict effectiveness of these agents, we may be wasting precious resources in trying to develop antiangiogenic agents further. New agents look attractive but intelligent clinical trial development will be required to find these new drugs a niche in an already saturated area of cancer treatment.
